# Behavioral changes following PCB 153 exposure in the Spontaneously Hypertensive rat – an animal model of Attention-Deficit/Hyperactivity disorder

**DOI:** 10.1186/1744-9081-10-1

**Published:** 2014-01-09

**Authors:** Espen Borgå Johansen, Frode Fonnum, Per L Lausund, S Ivar Walaas, Nora Elise Bærland, Grete Wøien, Terje Sagvolden

**Affiliations:** 1 Department of Physiology, Institute of Basic Medical Sciences, University of Oslo, Oslo, Norway; 2 Department of Biochemistry, Institute of Basic Medical Sciences, University of Oslo, Oslo, Norway; 3 Oslo and Akershus University College of Applied Sciences, Oslo, Norway; 4 The Norwegian Defense Research Establishment, Kjeller, Norway; 5 Department of Psychology, University of Oslo, Oslo, Norway

**Keywords:** ADHD, PCB, SHR, WKY, Behavior, Operant conditioning, Reinforcement

## Abstract

**Background:**

Attention-Deficit/Hyperactivity Disorder (ADHD) is a behavioral disorder affecting 3-5% of children. Although ADHD is highly heritable, environmental factors like exposure during early development to various toxic substances like polychlorinated biphenyls (PCBs) may contribute to the prevalence. PCBs are a group of chemical industrial compounds with adverse effects on neurobiological and cognitive functioning, and may produce behavioral impairments that share significant similarities with ADHD. The present study examined the relation between exposure to PCB 153 and changes in ADHD-like behavior in an animal model of ADHD, the spontaneously hypertensive rats (SHR/NCrl), and in Wistar Kyoto (WKY/NHsd) controls.

**Methods:**

SHR/NCrl and WKY/NHsd, males and females, were orally given PCB 153 dissolved in corn oil at around postnatal day (PND) 8, 14, and 20 at a dosage of 1, 3 or 6 mg/kg bodyweight at each exposure. The control groups were orally administered corn oil only. The animals were behaviorally tested for exposure effects from PND 37 to 64 using an operant procedure.

**Results:**

Exposure to PCB 153 was associated with pronounced and long-lasting behavioral changes in SHR/NCrl. Exposure effects in the SHR/NCrl depended on dose, where 1 mg/kg tended to reduce ADHD-like behaviors and produce opposite behavioral effects compared to 3 mg/kg and 6 mg/kg, especially in the females. In the WKY/NHsd controls and for the three doses tested, PCB 153 exposure produced a few specific behavioral changes only in males. The data suggest that PCB 153 exposure interacts with strain and sex, and also indicate a non-linear dose–response relation for the behaviors observed.

**Conclusions:**

Exposure to PCB 153 seems to interact with several variables including strain, sex, dose, and time of testing. To the extent that the present findings can be generalized to humans, exposure effects of PCB 153 on ADHD behavior depends on amount of exposure, where high doses may aggravate ADHD symptoms in genetically vulnerable individuals. In normal controls, exposure may not constitute an environmental risk factor for developing the full range of ADHD symptoms, but can produce specific behavioral changes.

## Background

Attention-Deficit/Hyperactivity Disorder (ADHD), a behavioral disorder affecting 3-5% of school-age children, is predominantly characterized by developmentally inappropriate patterns of inattention, hyperactivity, and impulsivity [1]. Results from studies of molecular genetics, pharmacological effects of stimulant drugs, and neuroimaging suggest that dopamine dysfunction is an important factor in ADHD etiology [2,3]. ADHD is a highly heritable disorder with an estimated heritability of ~0.76 [4]. Behavioral variance in heritability research is in a population often categorized into a genetic factor and an environmental factor. The gene-environment interaction (G × E), which is difficult to estimate, is usually incorporated into the genetic factor which inflates the estimate of purely genetic influences on ADHD prevalence and masks gene-environment interactions [5,6]. Thus, environmental factors may alone contribute to 20% of ADHD prevalence, and additionally may interact with a genetic vulnerability and produce ADHD [7]. Environmental risk factors identified thus far are cigarette smoking and alcohol use during pregnancy, and low birth weight [7-9]. Another environmental factor that either alone or in concert with a genetic vulnerability may contribute to ADHD prevalence is exposure during early development to various environmental toxic substances like lead, mercury or polychlorinated biphenyls (PCBs) [10-15].

PCBs are a group of chemical industrial compounds consisting of 209 congeners which have been used as oils and coolants in electrical equipment as well as in building material [16]. Of the estimated 1.2 million tons total production worldwide, 30% was discharged to the environment [17,18]. PCBs were banned around 1980 due to growing evidence that these compounds have adverse effects. They are chemically stable and resistant to degradation, and worldwide many areas continue to be heavily polluted. PCBs are stored in body-fat, accumulate in the food chain, and humans may be exposed prenatally both via trans-placental transfer and through breast milk during infancy. Adolescents and adults are mainly exposed through consumption of contaminated food, of which fish and seafood constitute the most important sources of PCB [16,17,19]. Studies show that even low-level exposure to PCBs during development has adverse effects on neurobiological, cognitive, and behavioral functioning [16,20,21]. Exposure may lead to impulsivity, reduced attention and concentration, poorer working memory and lower IQ scores [13,22-28], and give rise to behavioral impairments that share significant similarities with ADHD [15]. Studies have shown an association between PCB-levels in the umbilical cord blood and ADHD-behaviors as measured by Conners’ rating scale for teachers [13]. Moreover, an association between levels of cord serum PCBs and a higher omission rate on the Continuous Performance Test (CPT) and slower processing speed on WISC-III was found in boys, whereas for girls, the association was in the opposite direction for the CPT and zero for the WISC-III [29]. These latter findings suggest that exposure to PCBs may interact with gender to produce different behavioral and cognitive outcomes in males and females. Consistent with research on humans, studies show that PCB exposure affects learning and memory, activity level, and cognitive functions also in animals (for reviews, see [16,19]).

The dynamic developmental theory of ADHD [30] proposes that fundamental learning processes are altered in ADHD, consistent with suggestions of a reinforcement deficit in ADHD [31-41]. The dopamine system plays a major role in reinforcement and extinction of behavior which are the fundamental mechanisms of behavioral selection [42,43]. Behavior is selected by its consequences: Reinforcers increase the probability of behavior that produced them, and inadequate behavior is eliminated through the extinction process. From the selection and combination of simple behavioral units into longer behavioral sequences, habits and skills are built, and these behavioral sequences come under the control of environmental stimuli (stimulus control) as behavior has different consequences across environmental settings. According to the dynamic developmental theory of ADHD, dopamine dysfunction reduces the temporal window for associating antecedent stimuli and behavior with the behavior’s consequences (the three-term contingency), and weakens the elimination of inadequate behavior (extinction). These changes in fundamental learning processes are proposed to produce the behavioral symptoms of inattention (lack of stimulus-control), hyperactivity, and impulsivity (excess of responses with short inter-response times) observed in ADHD [44]. Additionally, the theory suggests that the sequencing of behavioral units into orderly, predictable chains of behavior is deficient in ADHD, producing short chains of behavior with overall low predictability. The predictability of behavioral chains in ADHD has been investigated using autocorrelations, where serial correlations between instances of behavior are calculated across the entire data set (e.g. the correlation between response n and response n + 1, between response n and response n + 2, …). These studies have found lower autocorrelations in the behavior of children with ADHD [45,46].

The present study examined the hypothesis that a relation exists between exposure to PCB 153 (2,2′,4,4′,5,5′-Hexachlorobiphenyl), a PCB congener found in human breast milk, and ADHD-like behavior in the spontaneously hypertensive rats (SHR/NCrl), an animal model of ADHD, and in Wistar Kyoto controls [47-49]. The SHR/NCrl represents a multifactorial genetic rat strain, and is with Wistar Kyoto (WKY/NHsd) controls a well validated animal model of ADHD [50,51]. Previous findings suggest that the behavioral changes observed in SHR/NCrl are linked to imbalanced dopamine and noradrenalin transmitter systems, both of which are affected by PCB exposure [48,52-56].

It was therefore hypothesized that the SHR/NCrl would be sensitive to PCB exposure to a greater extent than the WKY/NHsd controls. Findings suggest that PCB exposure activates compensatory brain mechanism [57,58], and a pre-existing transmitter system imbalance in SHR/NCrl may reduce the effectiveness of these compensatory brain mechanisms and lead to more behavioral changes following PCB exposure than in normal controls. We also wanted to explore if exposure would produce ADHD-like behaviors in WKY/NHsd control animals. In the present study, female and male animals were included to test if exposure effects were similar across sex, and short-term and long-term behavioral changes following exposure were investigated. Finally, three PCB doses were included to examine the dose–response relations in male and female SHR/NCrl and WKY/NHsd.

The rats were exposed to PCB 153 three times between postnatal day (PND) 8 and 20, which is a period of major brain development. In rats, the growth spurt of the brain peaks around PND 7 and is largely completed by PND 20. The corresponding time interval in humans is from birth to age 20, and must be considered when interpreting findings from translational research [59]. To accurately control dosing, the animals were in the present study exposed post-natally using a stomach tube, and effects of three doses (1, 3 or 6 mg/kg) representing low, medium, and high dose exposure were tested. Effects of PCB 153 exposure were subsequently assessed by reinforcer-controlled lever pressing representing operationalizations of deficient sustained attention (stimulus control), hyperactivity (lever presses with IRTs > 0.67 s), and impulsivity (responses with IRTs < 0.67 s). This behavioral procedure has been used in a number of previous studies of SHR/NCrl and WKY/NHsd including studies of drug-effects and PCB exposure [47,48,51,60,61]. As a supplement to lever-pressing, long-term effects of PCB exposure were in selected sessions assessed by video-recordings of the animal’s behavior during operant testing. Whenever the animal moved, the pixels changed from one video-frame to the next. These pixel-changes were used to calculate how amount of movement during testing. Additionally, we examined possible PCB exposure effects on the predictability of the sequential movement pattern by autocorrelating the animal’s positions in the operant chamber across the duration of the session. The behavioral testing of PCB exposure effects was completed before PND 70 to avoid the confounding factor of hypertension that SHR/NCrl develop from 10 to 12 weeks of age [62].

## Methods

### Subjects

Spontaneously hypertensive rats (SHR/NCrl) and Wistar Kyoto (WKY/NHsd) controls, males and females, were used. All animals were bred at the Norwegian Defense Establishment and the University of Oslo using breeders acquired from commercial breeding establishments: SHR/NCrl from Charles River, Germany, and WKY/NHsd from Harlan, England.

During the first three weeks, the rats were under the care of a veterinarian at the Norwegian Defense Research Establishment, Kjeller, Norway, who also administered the PCBs. The mother animals were caged singly under standard laboratory animal conditions (temperature ~22°C, humidity ~55%, 12 hr. light/dark cycle) in type IV macrolon cages and aspen bedding, where they also gave birth.

At PND 24, the rats were transported to the University of Oslo for behavioral testing. The rats were experimentally naïve on arrival. A total of 212 rats were behaviorally tested: 104 Spontaneously Hypertensive rats (SHR/NCrl) bred from SHR/NCrl breeders from Charles River, Germany, and 108 Wistar Kyoto rats (WKY/NHsd) bred from WKY/NHsd breeders from Harlan, England. Data from two control animals were excluded as the behavior was markedly deviant from the group mean and from previous control animal data, with z-scores around 3 on a number of consecutive sessions. Hence, a total number of 210 animals were included in the statistical analyses (Table 1).

**Table 1:** Number of subjects in the SHR/NCrl and WKY/NHsd PCB 153 exposure groups and control groups

	**Corn oil controls**		**PCB 1 mg/kg**		**PCB 3 mg/kg**		**PCB 6 mg/kg**	
	**Male**	**Female**	**Male**	**Female**	**Male**	**Female**	**Male**	**Female**
SHR	12	12	12	13	12	13	16	12
WKY	14	12	12	12	14	16	14	14

During habituation and response acquisition, the rats were housed together in twos or threes in 41 × 25 × 25 (height) cm transparent cages. Following acquisition of lever-pressing and throughout the rest of the study, the rats were housed individually in the same type of cages. The rats had free access to expanded pellet feed (RM3 (E) from Special Diet Services, Witham, Essex CM8 3 AD, UK) in the home cage at all times, and free access to water at all times prior to the dipper training sessions. Starting with the dipper training session and throughout the rest of the study, the rats were deprived of water for 21 hours a day.

The temperature in the housing area was ~22°C, and the light was on from 0700 to 1900 hours. The animals were tested once every day between 1000 and 1500 hours for a period of 40 days.

The study was approved by the Norwegian Animal Research Authority (NARA), and was conducted in accordance with the laws and regulations controlling experiments/procedures in live animals in Norway.

### Apparatus

The experimental procedure has been described previously [60,61]. Sixteen Campden Instruments operant chambers were used. The chambers were located in two separate rooms each of which contained eight chambers that were controlled by a separate computer. Each chamber was enclosed in a sound-resistant outer housing, was ventilated, and equipped with a grid floor. The animal’s working space in eight of the chambers was 25 × 25 × 30 (height) cm (room 1), and 25 × 25 × 20 (height) cm in the other eight chambers (room 2). A fan producing a low masking noise and a 2.8-W house light were on during the entire experimental session. Each chamber was equipped with two retractable levers requiring a dead weight of at least 3 g to activate a micro-switch, and with a 2.8-W cue light located above each lever.

The reinforcers (0.05 ml tap water) were delivered by a liquid dipper located in a small recessed cubicle where a 2.8-W cue light lit up when a reinforcer was presented. A 7 × 5 cm transparent plastic top-hinged flap separated the cubicle from the animal’s working space.

A computer program LabVIEW 7.1 recorded the behavior and scheduled reinforcers and lights [63].

A video camera manufactured by Tracer Technology Co., Ltd, Taiwan (Mini Color Hidden Cameras, 420TVL, 0, 1 lux) was installed in each operant chamber. The camera was positioned in the upper rear corner of the ceiling at an angle of 45°, and was controlled by the VR Live Capture computer program (Novus Security, Warsaw, Poland) which saved the video-files (15 frames/s) for analyses.

### Procedure

#### PCB exposure

The animals were assigned to one of the three experimental groups or to the control group and then orally given one of the three doses of PCB 153 (2,2′,4,4′,5,5′-hexachlorobiphenyl) dissolved in corn oil, or corn oil only (Table 1). Experimentation took place over an extended period of time, with different doses of PCB 153 tested at different times. The PCB 153 was purchased from Patrick Anderson, Department of Chemistry, University of Umeå, Sweden, and was specially purified and free from dioxin-like PCBs. At each exposure, the dosage used was either 1, 3 or 6 mg/kg bodyweight with a total volume of 0.01 ml/g body weight administered by gavage with a stomach tube. As the exact time of birth was not observed in all cases (e.g. during night-time) and exposure was performed by the veterinarian during working hours, the three PCB exposures took place at around PND 8, PND 14, and PND 20 with minor variations around these ages.

#### Habituation, dipper training, and response acquisition

Prior to behavioral testing, the rats were assigned an operant chamber and a time of testing in a semi-randomized and balanced way. Habituation to the operant chambers started at the day following arrival (PND 25) and lasted 30 min. During the habituation session, the flap between the working space and the reinforcement cubicle was taped open. No levers were present, the cue lights above the levers were off, and no reinforcers were delivered.

The habituation session was followed by two 30-min dipper training sessions. The flap was taped open, no levers were present, and the cue lights above the levers were off. The computer delivered water every 10 s independent of the animal’s behavior using a fixed-time schedule of reinforcement. The cue light in the small recessed cubicle was turned on during each water delivery, and the reinforcer was available for 3 seconds.

In the following two sessions, the animals were trained to open the flap to gain access to the drop of water. The tape was removed from the flap, no levers were present, and the cue lights located above the levers were off. Each flap-opening turned on the cue light in the water cubicle and produced the presentation of a single drop of water. The water-dipper was lowered after 5 s irrespective of the animal’s behavior.

During the subsequent two sessions, lever-pressing was shaped according to the method of successive approximations [64]. During the first of these sessions, the animals learned to press the left lever in order to receive a reinforcer immediately following every press. The cue light above the left lever was lit for the entire session except during presentation of the reinforcer when the light in the water cubicle was turned on. During this session, the right lever was retracted into the wall and the light above the lever was off. On the second session, the right lever was inserted and the left lever was retracted. The light above the right lever was lit the entire session except during presentation of the reinforcer when the light in the water cubicle was turned on. Immediately following response shaping on each lever, the animal was monitored to make sure the response was learned, and then left in the chamber for an additional 15 min to further strengthen the newly learned behavior. During this time, every press on the lever produced a reinforcer.

#### The variable interval 3 s schedule

Response acquisition was followed by five 30-min long training sessions (sessions 8–12) using a variable interval (VI) 3 s reinforcement schedule. During the VI 3 s sessions and throughout the rest of the study, both levers were present. At the start of the session and following each reinforcer delivery, the computer program semi-randomly selected which lever produced the reinforcer. Lever selection was limited to a maximum of 4 consecutive reinforcers on the same lever to avoid the development of lever-preference.

The lever producing the reinforcer was signaled by the lit cue light (discriminative stimulus) located above the lever. The light stayed lit for as long as the lever was associated with reinforcement, but was turned off during reinforcer presentation. The timer for the next interval started when the dipper was presented. Scheduled reinforcers and reinforcers produced, but not collected, were accumulated and scheduled for the next correct response.

Except for during the habituation and dipper training sessions, reinforcers were accessible for 3 s after the flap into the water cubicle was opened. Then, the dipper was lowered and the cubicle light was turned off. If the flap was not opened within 5 s after a reinforcer presentation, the water dipper was lowered and the cubicle light was turned off.

A concurrent extinction schedule was in effect on the alternative lever. The light above the alternative lever was always off. Thus, the present task can be described as a simultaneous visual discrimination task.

#### The VI 180 s schedule

A variable interval 180 s schedule (VI 180 s) was in effect for 90 min on one of the two levers from session 13 to session 40 (see Table 2 for a summary of the experimental procedure). A computer program was used to generate a Catania-Reynolds distribution of intervals for the VI 180 s schedule [65,66]. Inter-reinforcer intervals during the VI 180 s schedule ranged from 6 s to 719 s and were distributed in a semi-randomized fashion across the session. There was neither any external stimulus signaling that a reinforcer was programmed nor any external stimulus signaling the time since the last response.

**Table 2:** Overview of the experimental procedure

**Session number**	**Schedule**	**Notes**
1		Habituation
2 – 3	FT 10 s	Magazine training
4 – 5	CRF	Flap training
6 – 7		Shaping of lever pressing
8 – 12	VI 3 s	30 min session
13 – 40	VI 180 s	90 min session

*Note*. FT = Fixed time; CRF = continuous reinforcement; VI = Variable interval.

#### Behavioral measures - lever pressing

The computer recorded lever presses on the two levers, flap openings to the cubicle, reinforcers produced and collected, and the time of the events. Stimulus control (sustained attention), hyperactivity, and responses with interresponse-times < 0.67 s (impulsivity) were calculated the following way [60,61]:

##### Stimulus control - sustained attention

Stimulus control, the percentage of presses on the correct lever, was used as a measure of sustained attention. To produce a reinforcer, the animal had to pay attention to and press the correct lever as signaled by the lit cue light located above the lever. If the animals paid attention to the light and mostly pressed the correct lever, percentage correct would be high. Percentage correct would be at chance level (~50%) in animals not paying attention to the cue light and pressing both levers equally often.

##### Activity - IRTs > 0.67 s

Inter-response times (IRTs), the time interval between two consecutive lever presses, were recorded and divided into short IRTs (< 0.67 s) and long IRTs (> 0.67 s). Level of activity was measured as the total number of lever presses with IRTs > 0.67 s on the correct and wrong levers combined.

##### Impulsivity - IRTs < 0.67 s

Responses with IRTs shorter than 0.67 s were used as a measure of impulsivity (“premature responding” or “inability to wait”).

#### Behavioral measures - video-recordings

In selected sessions, the animals were video-recorded during the operant testing. The computer program Musical Gestures Toolbox, developed for audio and video analysis [67], analyzed frame-to-frame changes in pixels which occurred whenever the animal moved. Pixel changes were averaged across 15 frames, and a noise reduction threshold and a filter were used to clarify the motion images and improve the analyses. Animal movement produced a sphere of pixels that changed from frame to frame. Amount of movement (Quantity of Motion, QoM) was calculated as the ratio of changed pixels relative to total number of pixels on the screen (QoM is 0 when there is no movement and no pixels change from frame to frame, and 1 if all pixels change). The center of the sphere was used to calculate the position of the animal (x and y positions). Serial correlations (autocorrelations) of the x and y positions were calculated across the session and were used to analyze predictability of movement in the test chamber. The correlation between position at time t and position at time t + 1 represents lag 1, the correlation between position at time t and position at time t + 2 represents lag 2, and so forth.

### Data analysis

All statistical analyses were done in Statistica 6.0 [68]. Data were evaluated by multivariate analyses using Wilks lambda (MANOVAs) when the degrees of freedom relative to the number of levels of the repeated factor permitted this approach, or by univariate analyses of variance (ANOVAs) adjusting the degrees of freedom with the Greenhouse-Geisser epsilon [69]. Post-hoc tests were performed using the Unequal N HSD test for analysis of unequal sample sizes [68]. Missing data were substituted by calculating the means of the preceding and following sessions. Outliers were identified by calculating z-scores for each individual group, and data with z-scores exceeding ± 3 were excluded from the analyses.

Analyses of variance assume that data are normally distributed. Although ANOVAs may be relatively robust against such violations, inflated Type I and II error rates may be the result if the normal distribution requirement is not met. In the analyses of lever-pressing, 3 between subjects factors were analyzed: strain (2 levels), sex (2 levels), and dose (4 levels), producing a total of 16 subgroups. The data distributions for lever-pressing in these subgroups for each session were analyzed for identification of normal distribution violations using Shapiro-Wilk tests, and several violations were found. In order to normalize data, arc sine, log10, and square root transformations of the data were performed and evaluated by number of variables normalized. Square root transformations produced the best results, and consequently, all data were square root transformed prior to the statistical analyses except for autocorrelation data.

#### Lever presses and reinforcers collected

Measures cumulated across the session were used in the analyses. The first 7 sessions under VI 180 s were analyzed separately for short-term behavioral changes following PCB exposure. In a second analysis, the remaining 21 sessions were analyzed for long-term changes. Sessions were used as the within-subject factor, and strain, sex, and dose of PCB-exposure were used as between-subjects factors.

#### Video-recordings

Video-recordings from sessions 30 and 39 under the VI 180 s schedule were analyzed together. These two sessions were chosen to investigate long-term changes in video-recorded behavior: Quantity of motion (QoM) and autocorrelations of the x-y positions in the operant chamber.

For quantity of motion, data averaged across the 90 min session were used in the analyses. Sessions were used as the within-subject factor, and strain, sex, and dose of PCB-exposure were used as between-subjects factors.

Video-recording of the entire session were used to calculate 42 autocorrelation lags for the x- and y-position in the operant chamber. Preliminary analyses showed similar results for autocorrelations of the x- and y-positions. To limit the number of independent variables and simplify the analyses, these two measures were therefore averaged into one xy-position variable before the statistical analyses. Sessions and lags were used as the within-subject factors, and strain, sex, and dose of PCB-exposure were used as between-subjects factors.

## Results

**Figure 1:**
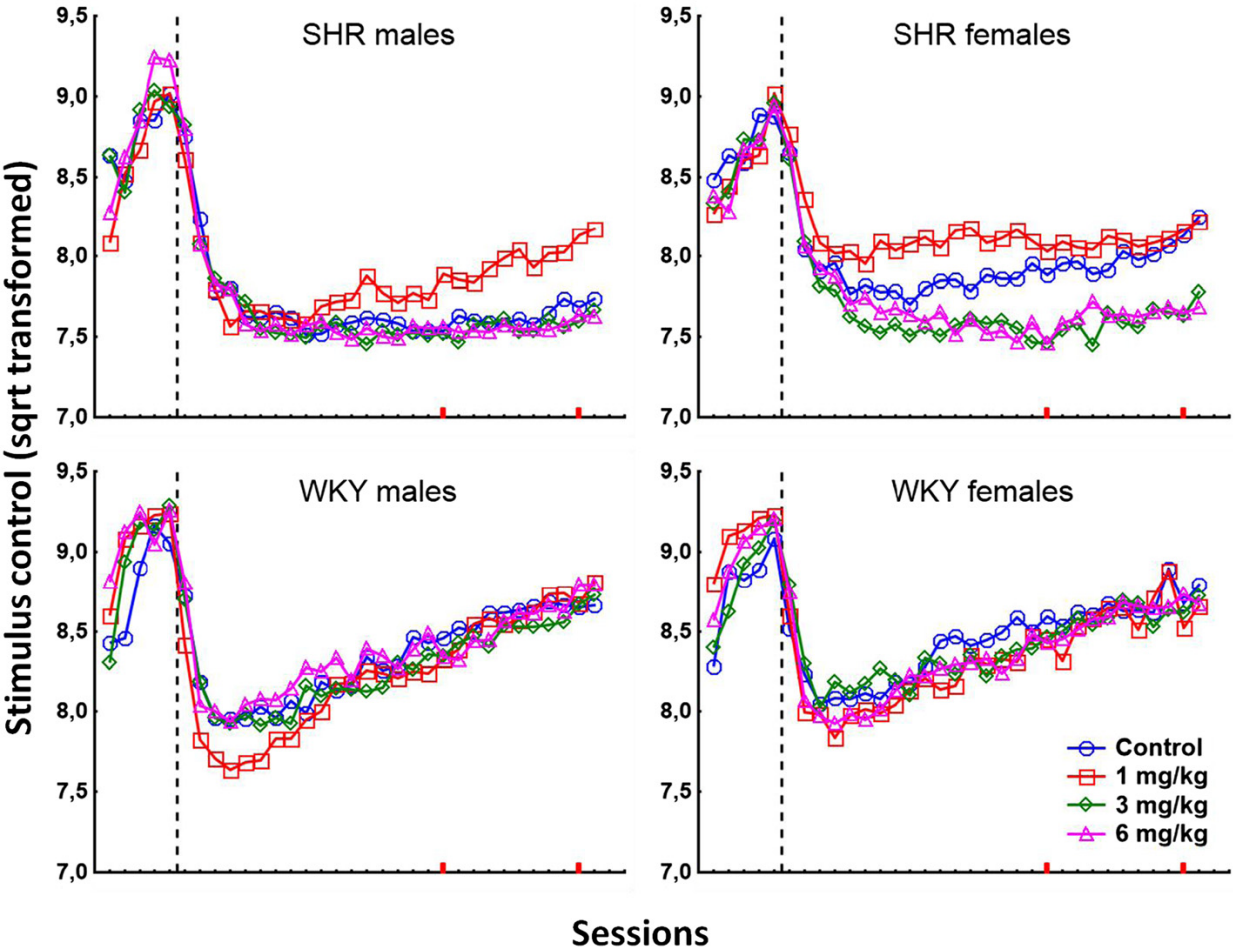
**Stimulus control (square root-transformed percentage of responses on the reinforcer-producing lever) in SHR/NCrl and WKY/NHsd, males and females, exposed to 1, 3, or 6 mg/kg PCB 153 or to corn oil only.** A percentage of √50 (~7.07) represents chance level where the animal presses the reinforcer-producing lever and the alternative the same number of times. The stippled lines represent the change in reinforcement schedule from VI 3 s to VI 180 s. Sessions 30 and 39, used in the video-analyses, are marked on the x-axes in red.

**Figure 2:**
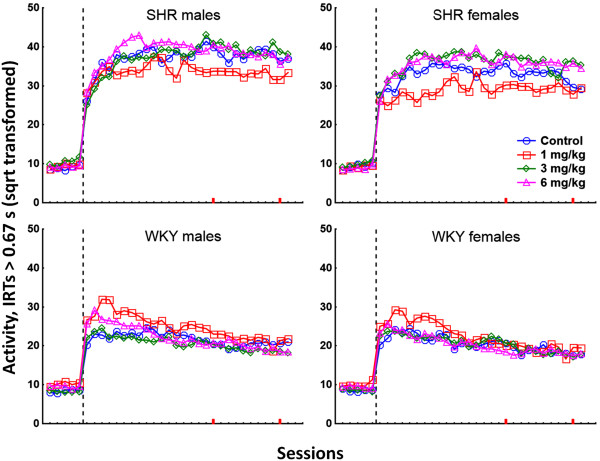
**Lever pressing with long IRTs (square root-transformed responses with IRTs > 0.67 s) in SHR/NCrl and WKY/NHsd, males and females, exposed to 1, 3, or 6 mg/kg PCB 153 or to corn oil only.** The stippled lines represent the change in reinforcement schedule from VI 3 s to VI 180 s. Sessions 30 and 39 used in the video-analyses are marked on the x-axes in red.

**Figure 3:**
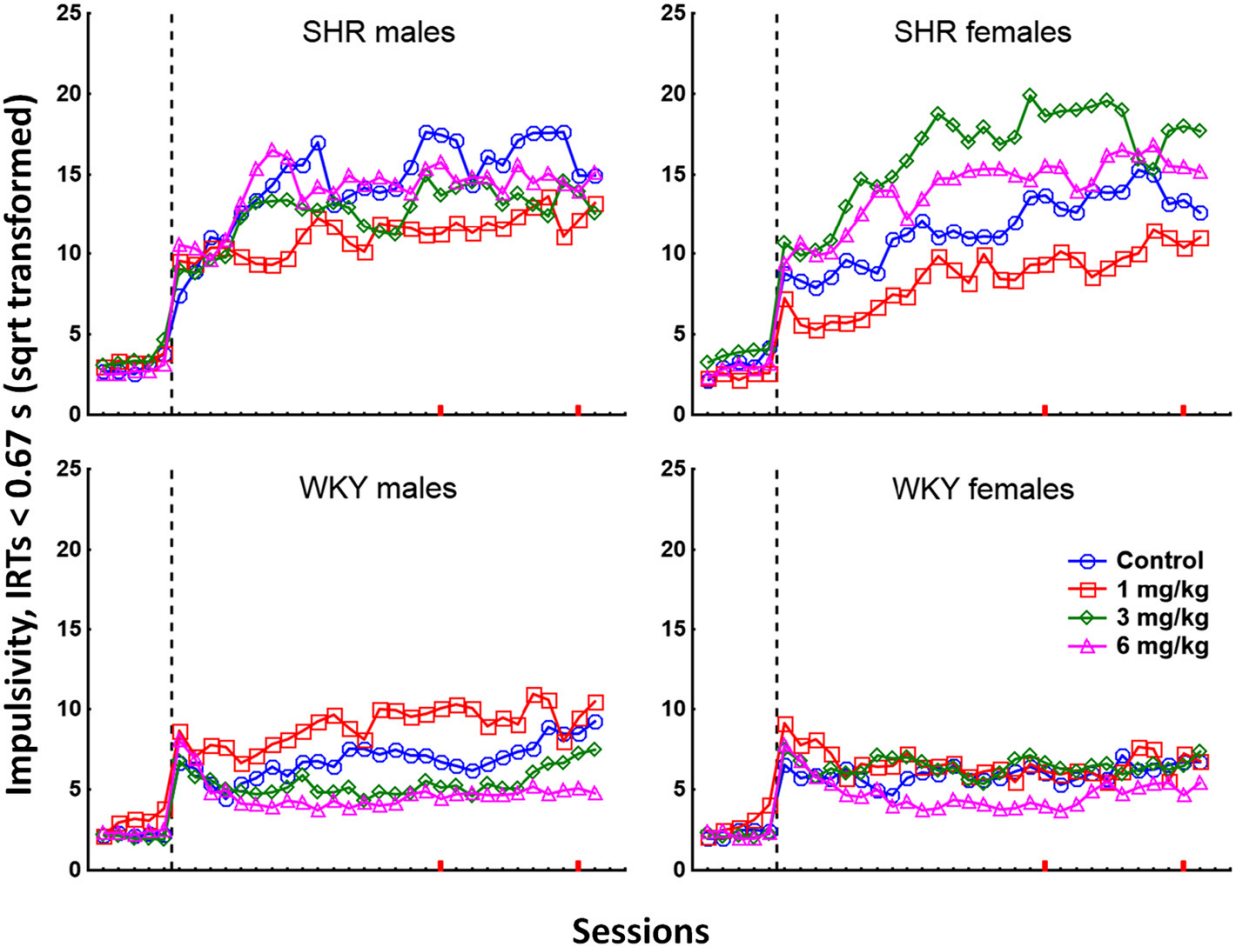
**Lever pressing with short IRTs (square root-transformed responses with IRTs < 0.67 s) in SHR/NCrl and WKY/NHsd, males and females, exposed to 1, 3, or 6 mg/kg PCB 153 or to corn oil only.** The stippled lines represent the change in reinforcement schedule from VI 3 s to VI 180 s. Sessions 30 and 39 used in the video-analyses are marked on the x-axes in red.

**Figure 4:**
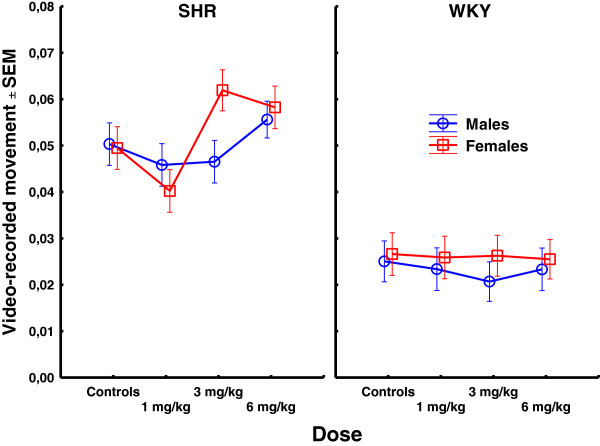
**Video-recorded movement (square root-transformed ratio of frame-to-frame pixels-change) across sessions 30 and 39 in SHR/NCrl and WKY/NHsd, males and females, exposed to 1, 3, or 6 mg/kg PCB 153 or to corn oil only.** The data suggest a non-linear dose–response relationship in SHR/NCrl females.

**Figure 5:**
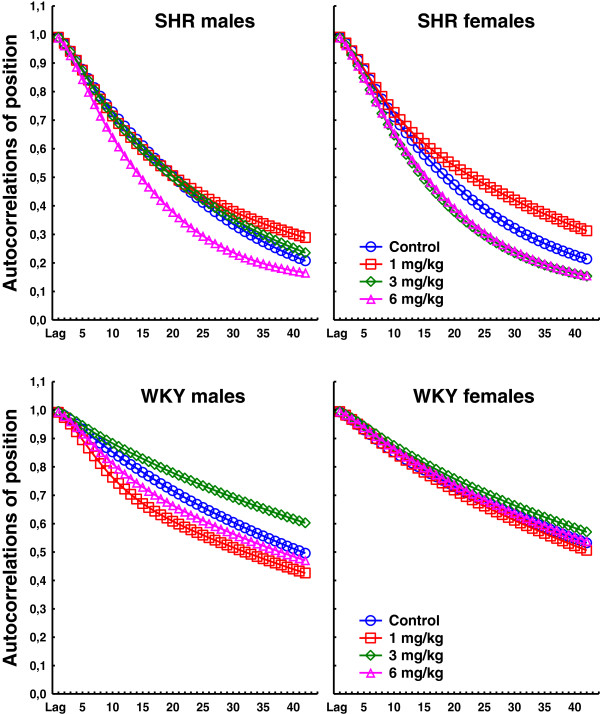
**Autocorrelations of the xy-position in the operant chamber in SHR/NCrl and WKY/NHsd, males and females, exposed to 1, 3, or 6 mg/kg PCB 153 or to corn only during sessions 30 and 39.** The data suggest a non-linear dose–response relationship that interacts with strain and sex.

PCB exposure produced a complex pattern of behavioral effects in the two strains. Data from all sessions for stimulus control, activity, and impulsivity are presented in Figures 1, 2, and 3, respectively. Data from video-analyses of amount of movement and autocorrelations of the position in the operant chamber are presented in Figures 4 and 5, respectively. In general, the observed effects were most pronounced in SHR/NCrl, but some changes were also observed in the WKY/NHsd controls. The time course of effects was different in SHR/NCrl and WKY/NHsd, and somewhat different for males and females. Additionally, relative to the control condition, in the SHR/NCrl there was a tendency for the lowest PCB-dose (1 mg/kg) to have opposite effects compared to the highest dose (6 mg/kg).

### Lever presses

#### Stimulus control (sustained attention) during the first 7 sessions

The analyses showed a significant main effect of rat strain, F(1,194 = 17.58; p < 0.001), with better stimulus control in WKY/NHsd than in SHR/NCrl. Stimulus control was overall better in females than males, and there was a statistically significant main effect of sex, F(1,194 = 8.42; p < 0.01).

Stimulus control decreased across sessions, F(6,189 = 172.62; p < 0.001), less so in WKY/NHsd than in SHR/NCrl, and less in females than in males. The analyses showed statistically significant strain × session, F(6,189 = 11.59; p < 0.001), and sex × session, F(6,189 = 2.24; p < 0.05), interaction effects. There was also a statistically significant dose × session interaction effect, F(18,535.06 = 2.20; p < 0.01).

##### Strain × dose interaction effect

The analyses of the first 7 sessions showed a statistically significant strain × dose interaction effect, F(3,194 = 5.11; p < 0.01). Unequal N HSD post hoc tests of this effect showed that stimulus control was significantly lower in WKY/NHsd exposed to 1 mg/kg than in WKY/NHsd exposed to 3 mg/kg (*p* < 0.05). No statistically significant differences between exposure conditions were found in SHR/NCrl.

#### Stimulus control (sustained attention) during the last 21 sessions

The analyses showed that stimulus control was generally better in WKY/NHsd than in SHR/NCrl, and overall better in females than in males. The analyses showed statistically significant main effects of strain, F(1,192 = 198.80; p < 0.001), and sex, F(1,192 = 5.11; p < 0.05). There was a statistically significant main effect of dose, F(3,192 = 3.70; p < 0.05), where stimulus control was highest in animals exposed to 1 mg/kg, and better in controls than in animals exposed to 3 mg/kg and 6 mg/kg. Stimulus control increased across the final 21 sessions, F(20,173 = 16.46; p < 0.001). This increase was steeper in WKY/NHsd than in SHR/NCrl where little change in stimulus control across sessions was observed. The strain × session interaction effect was statistically significant, F(20,173 = 5.93; p < 0.001). There was also a statistically significant dose × session interaction effect, F(60,516.97 = 1.48; p < 0.05), where stimulus control was better in animals exposed to 1 mg/kg than in controls, and better in controls than in animals exposed to 3 mg/kg and 6 mg/kg.

##### Strain × dose interaction effect

The analyses of the final 21 sessions showed a statistically significant strain × dose interaction effect, F(3,192 = 4.17; p < 0.01). Unequal N HSD post hoc tests showed that stimulus control was significantly better in SHR/NCrl exposed to 1 mg/kg relative to SHR/NCrl exposed to 3 mg/kg and 6 mg/kg (*p* < 0.001 and *p* < 0.01, respectively). No statistically significant differences between exposure conditions were found in WKY/NHsd.

#### Responses with IRTs > 0.67 s (activity) during the first 7 sessions

SHR/NCrl emitted more responses with IRTs > 0.67 s than WKY/NHsd. Overall, males produced more responses than females. The analyses showed statistically significant main effects of strain, F(1,192 = 192.71; p < 0.001), and sex, F(1,192 = 9.90; p < 0.01). The analyses also showed a statistically significant main effect of dose, F(3,192 = 2.94; p < 0.05), with fewer responses produced by unexposed animals than by animals exposed to 6 mg/kg, and with intermediate levels of responding in the 1 mg/kg and 3 mg/kg exposure groups. Number of responses increased across sessions, F(6,187 = 32.84; p < 0.001). This increase was mainly produced by the SHR/NCrl, and there was a statistically significant strain × session interaction effect, F(6,187 = 18.79; p < 0.001). The increase in responding across sessions was steeper in males than in females, and less steep in animals exposed to 1 mg/kg than in the other exposure group, and the analyses showed statistically significant sex × session, F(6,187 = 2.27; p < 0.05), and dose × session, F(18,529.40 = 3.96; p < 0.001), interaction effects.

##### Strain × dose interaction effect

The analyses showed a statistically significant strain × dose interaction effect, F(3,192 = 11.33; p < 0.001). Unequal N HSD post hoc analyses showed that SHR/NCrl exposed to 1 mg/kg produced significantly fewer responses than SHR/NCrl exposed to 6 mg/kg (*p* < 0.001). For WKY/NHsd, animals exposed to 1 mg/kg produced significantly more responses than unexposed animals and animals exposed to 3 mg/kg (*p*s < 0.001).

##### Strain × dose × session interaction effect

SHR/NCrl exposed to 1 mg/kg produced fewer responses than SHR/NCrl in the other exposure conditions. In WKY/NHsd, more responses were produced during the first sessions by animals exposed to 1 mg/kg relative to WKY/NHsd in the other exposure conditions, and the analyses showed a statistically significant strain × dose × session interaction effect, F(18,529.40 = 2.46; p < 0.05).

#### Responses with IRTs > 0.67 s (activity) during the last 21 sessions

SHR/NCrl emitted more responses with IRTs > 0.67 s than WKY/NHsd, and more responses were emitted by males than females. The analyses showed statistically significant main effects of strain, F(1,189 = 665.63; p < 0.001), and sex F(1,189 = 18.29; p < 0.001). Number of responses with IRTs > 0.67 s decreased across sessions, and this main effects was statistically significant F(20,170 = 11.73; p < 0.001). This decrease was more pronounced in WKY/NHsd than in SHR/NCrl, and the analyses showed a statistically significant strain × session interaction effect, F(20,170 = 6.91; p < 0.001). The decrease in responding across sessions was different for the exposure groups, and most pronounced in animals exposed to 1 mg/kg. The analyses showed a statistically significant dose × session interaction effect, F(60,508.02 = 5.27; p < 0.001).

##### Strain × dose interaction effect

Analyses of IRTs > 0.67 s across the last 21 sessions showed a statistically significant strain × dose interaction effect, F(3,189 = 11.69; p < 0.001). For SHR/NCrl, unequal N HSD post hoc analyses showed that animals exposed to 1 mg/kg produced significantly fewer responses than SHR/NCrl controls and animals exposed to 3 mg/kg and 6 mg/kg (*p* < 0.05, *p* < 0.001, *p* < 0.001, respectively). No statistically significant differences were found across exposure conditions in WKY/NHsd.

##### Strain × dose × session interaction effect

In SHR/NCrl, animals exposed to 1 mg/kg produced fewer responses than animals in the other exposure conditions, and this difference was stable across sessions. In WKY/NHsd, animals in the 1 mg/kg exposure group produced more responses during the first sessions than animals in the other exposure conditions, whereas this difference was small at the end of testing. This strain × dose × session interaction effect was statistically significant, F(60,508.02 = 3.06; p < 0.001).

#### Responses with IRTs < 0.67 s (impulsivity) during the first 7 sessions

SHR/NCrl produced more responses with IRT < 0.67 s than WKY/NHsd, and the analyses showed a statistically significant main effect of strain, F(1,189 = 97.70; p < 0.001. The number of short IRTs increased across the first 7 sessions as shown by the statistically significant main effect of sessions, F(6,184 = 9.10; p < 0.001. However, whereas number of short IRTs increased in SHR/NCrl, the number decreased in WKY/NHsd, and the analyses showed a statistically significant strain × session interaction effect, F(6,184 = 14.60; p < 0.001. The number of short IRTs increased across sessions in all exposure groups except in animals exposed to 1 mg/kg, and there was a statistically significant dose × session interaction effect, F(18,520.92 = 2.93; p < 0.001.

##### Strain × sex and sex × dose interaction effects

The analyses showed a statistically significant strain × sex interaction effect, F(1,189 = 6.69; p < 0.05. Male SHR/NCrl produced more responses with IRTs < 0.67 s than SHR/NCrl females whereas the numbers of short IRTs were similar across sexes in WKY/NHsd. This was confirmed by unequal N HSD post hoc tests showing a significant difference between SHR/NCrl males and females (*p* < 0.05) while no significant differences were found for WKY/NHsd.

The number of short IRTs produced by females varied across exposure conditions, with the lowest and highest numbers of responses produced by the 1 mg/kg and 3 mg/kg exposure groups, respectively. Males produced comparable numbers of short IRTs across the exposure conditions. This sex × dose interaction effect was statistically significant, F(3,189 = 3.15; p < 0.05. Unequal N HSD post hoc test of this effect showed that females exposed to 1 mg/kg produced significantly fewer responses than females exposed to 3 mg/kg (*p* < 0.05), whereas no significant effects were found in males.

##### Strain × dose interaction effect

The analyses showed a statistically significant strain × dose interaction effect, F(3,189 = 8.79; p < 0.001. Unequal N HSD post hoc analyses showed that for SHR/NCrl, animals exposed to 1 mg/kg produced significantly fewer responses with IRTs < 0.67 s than animals exposed to 3 mg/kg and 6 mg/kg (*p*s < 0.001). No significant effects were found across exposure conditions in WKY/NHsd.

##### Strain × dose × session interaction effect

The number of short IRTs produced by SHR/NCrl exposed to 1 mg/kg was stable across sessions but increased across session in all other SHR/NCrl exposure groups. In WKY/NHsd, there was as small decrease in number of short IRTs in all exposure conditions. The analyses showed a statistically significant strain × dose × session interaction effect, F(18,520.92 = 1.91; p < 0.05.

#### Responses with IRTs < 0.67 s (impulsivity) during the last 21 sessions

The analyses of the last 21 sessions showed a main effect of strain, F(1,184 = 170.51; p < 0.001, with SHR/NCrl emitting more responses with short IRTs than WKY/NHsd. The number of responses with short IRTs increased across sessions as reflected in a statistically significant main effect of session, F(20,165 = 4.98; p < 0.001. This increase was more pronounced in SHR/NCrl than in WKY/NHsd and was different across exposure conditions, and the analyses showed statistically significant strain × session, F(20,165 = 3.08; p < 0.001, and dose × session, F(60,493.10 = 3.44; p < 0.001, interaction effects. In SHR/NCrl, the number of produced short IRTs increased more steeply across sessions in females than in males whereas the opposite was observed in WKY/NHsd, and the analyses showed a statistically significant strain × sex × session interaction effect, F(20,165 = 2.00; p < 0.01.

##### Strain × dose and sex × dose interaction effects

In SHR/NCrl, the number of short IRTs was stable across exposure conditions except in the 1 mg/kg exposure group where a reduction in number of short IRTs was observed. In WKY/NHsd, number of short IRTs was similar across exposure conditions. This strain × dose interactions effect was statistically significant, F(3,184 = 8.04; p < 0.001. Unequal n HSD post hoc analyses showed that in SHR/NCrl, animals exposed to 1 mg/kg produced fewer responses than animals exposed to 3 mg/kg and 6 mg/kg (*p* < 0.01 and *p* < 0.05, respectively), and non-significantly fewer responses than unexposed controls (*p* = 0.058). No effects of exposure were found in WKY/NHsd.

The analyses also showed a statistically significant sex × dose interaction effect, F(3,184 = 4.21; p < 0.01. In males, the observed number of short IRTs decreased across exposure conditions, whereas females exposed to 3 mg/kg produced more responses than the other female exposure groups. Unequal N HSD post hoc tests of the sex × dose interaction effect showed no statistically significant effects.

##### Strain × dose × session interaction effect

In SHR/NCrl, the observed number of responses with short IRTs was lowest in the 1 mg/kg exposure group and increased across sessions, whereas the number was similar across sessions in the other SHR/NCrl exposure groups. WKY/NHsd exposed to 1 mg/kg produced a stable number of responses with short IRTs across sessions that was higher compared to the other exposure groups, and especially compared to the 6 mg/kg exposure group where the lowest number of responses with short IRTs was observed. The analyses showed a statistically significant strain × dose × session interaction effect, F(60,493.10 = 2.43; p < 0.001.

#### Reinforcers collected

The majority of the possible 30 reinforcers available during each session under the VI 180 s schedule were produced and collected by the animals during testing. The statistical analyses showed significant group differences in the number of reinforcers collected. However, these were small and unlikely to explain the behavioral differences observed in the study.

Analyses of the first 7 sessions under VI 180 s showed that the number of reinforcers collected increased across sessions as reflected in a statistically significant main effect of session, F(6,189) = 2.77; p < 0.05. The observed number of reinforcers collected was marginally higher in WKY/NHsd than in SHR/NCrl during 5 of the sessions, and the analyses showed a statistically significant strain × session interaction effect, F(6,189) = 2.41; p < 0.05. Unequal N HSD post-hoc analyses of the strain × session interaction effect showed that the SHR/NCrl in session 1 collected significantly fewer reinforcers than the WKY/NHsd in session 7 (*p* < 0.05), whereas no strain differences were found for same-session comparisons. Strain mean was 28.50 in SHR/NCrl, while strain mean for WKY/NHsd was 28.24. The number of reinforcers collected in the subgroups ranged from 28.04 in WKY/NHsd control females to 28.56 in SHR/NCrl males exposed to 6 mg/kg.

Analyses of reinforcers collected during the final 21 sessions showed a statistically significant main effect of strain, F(1,193) = 27.00; p < 0.001, a significant main effect of session, F(20,174) = 2.05; p < 0.01, and a significant dose × session interaction effect, F(60,519.96) = 1.47; p < 0.05. Again, differences were small: Strain mean was 28.27 in SHR/NCrl and 28.18 in WKY/NHsd, and ranged between subgroups from 27.88 in WKY/NHsd male controls to 28.52 in SHR/NCrl males exposed to 1 mg/kg. Unequal N HSD post-hoc analyses of the dose × session interaction effect showed that in one of the 21 sessions analyzed (sessions 7), significantly more reinforcers were collected by animals exposed to 1 mg/kg (mean = 28.57) than animals exposed to 3 mg/kg (mean = 27.46) (*p* < 0.05). No other significant differences were found across doses for same-session comparisons.

### Video analyses

#### Quantity of motion

Amount of video-recorded movement was generally higher in SHR/NCrl than in WKY/NHsd, and higher in females than in males, and the analyses showed statistically significant main effects of strain, F(1,187) = 541.24; p < 0.001, and sex, F(1,187) = 6.63; p < 0.05. Amount of video-recorded movement was different across exposure conditions, and the analyses showed a statistically significant main effect of dose, F(3,187) = 6.42; p < 0.001. Unequal N HSD post hoc tests showed that animals exposed to 1 mg/kg moved significantly less than animals exposed to 3 mg/kg or 6 mg/kg (*p* < 0.05 and *p* < 0.001, respectively). Amount of movement increased across sessions, F(1,187) = 8.85; p < 0.01, but only in WKY/NHsd, and there was a statistically significant strain × session interaction effect, F(1,187) = 4.28; p < 0.05. The analyses also showed at statistically significant dose × session interaction effect, F(3,187) = 10.49; p < 0.001, where amount of movement increased across sessions in all but the 6 mg/kg exposure group.

##### Strain × dose interaction effect

The analyses showed a statistically significant strain × dose interaction effect, F(3,187) = 8.06; p < 0.001. Post hoc tests showed that SHR/NCrl exposed to 1 mg/kg moved less than SHR/NCrl exposed to 3 mg/kg or 6 mg/kg (*p*s < 0.001) whereas no effects of exposure were found in WKY/NHsd.

##### Sex × dose interaction effect

In males, amount of movement was a u-shaped function of PCB dose whereas in females this function was serpentine-shaped. The sex × dose interaction effect was statistically significant, F(3,187) = 5.45; p < 0.01. Post hoc tests showed that males exposed to 1 mg/kg or 3 mg/kg moved significantly less than males exposed to 6 mg/kg (*p* < 0.05 and *p* < 0.01, respectively) whereas females exposed to 1 mg/kg moved less than females exposed to 3 mg/kg and 6 mg/kg (*p* < 0.001 and *p* < 0.05, respectively).

##### Strain × sex × dose interaction effect

The non-linear relation between movement and PCB exposure in males and females were observed in SHR/NCrl only whereas no exposure effects on movement were observed in WKY/NHsd. The strain × sex × dose interaction effect was statistically significant, F(3,187) = 2.70; p < 0.05. Unequal N HSD post-hoc analyses of this 3-way interaction effect showed that unexposed SHR/NCrl females moved less than SHR/NCrl females exposed to 3 mg/kg (*p* < 0.05), and that SHR/NCrl females exposed to 1 mg/kg moved less than SHR/NCrl females exposed to 3 mg/kg or 6 mg/kg (*p*s < 0.001). No other effects were found.

#### Autocorrelations of the animals’ in the operant chamber

The autocorrelations of the animals’ positions in the operant chamber were generally higher in WKY/NHsd than in SHR/NCrl as reflected in a statistically significant main effect of strain, F(1,185) = 260.15; p < 0.001. The analyses also showed a main effect of dose, F(3,185) = 2.92; p < 0.05, with lower autocorrelations in animals exposed to 6 mg/kg. The autocorrelations declined across lags, and there was a statistically significant main effect of lag, F(1.60,295.58) = 4108.16; p < 0.001.

##### Session interaction effects

The observed autocorrelations increased across the two sessions in SHR/NCrl whereas a decrease in autocorrelations was observed in WKY/NHsd. The observed autocorrelations in females were higher than in males during session 30 and similar to males during session 39. The analyses showed statistically significant strain × session, F(1,185) = 9.74; p < 0.01, and sex × session, F(1,185) = 5.24; p < 0.05, interaction effects. Post hoc tests of these effects showed no significant differences in addition to those reflected in the main effects. The autocorrelations were lower during session 30 in animals exposed to 6 mg/kg compared to the 1 mg/kg and 3 mg/kg exposure groups, and the analyses showed a statistically significant dose × session interaction effect, F(3,185) = 3.66; p < 0.05.

##### Lag interaction effects

The autocorrelations were lower and declined more steeply across lags in SHR/NCrl than in WKY/NHsd, and the analyses showed a statistically significant strain × lag interaction effect, F(1.60,295.58) = 261.40; p < 0.001. In SHR/NCrl, the autocorrelations declined marginally steeper across lags during session 30 than during session 39 whereas the opposite was observed in WKY/NHsd. The analyses showed a statistically significant strain × session × lag interaction effect, F(1.42,262.63) = 6.08; p < 0.01. Relative to the other exposure conditions, the autocorrelations were generally lower and declined more steeply across lags in animals exposed to 6 mg/kg, especially during session 30, and the analyses showed statistically significant dose × lag, F(4.79,295.58) = 3.77; p < 0.01, and dose × session × lag, F(4.26,262.63) = 3.65; p < 0.01, interaction effects.

##### Strain × dose and sex × dose interaction effects

The analyses showed a statistically significant strain × dose interaction effect, F(3,185) = 7.21; p < 0.001. Post hoc analyses of this effect showed that the autocorrelations in SHR/NCrl exposed to 1 mg/kg were significantly higher than in SHR/NCrl exposed to 6 mg/kg (*p <* 0.001) whereas the autocorrelations in WKY/NHsd exposed to 1 mg/kg were significantly lower than in WKY/NHsd exposed to 3 mg/kg (*p* < 0.05).

The analyses also showed a statistically significant sex × dose interaction effect, F(3,185) = 3.03; p < 0.05. Post hoc analyses showed that the autocorrelations in males exposed to 3 mg/kg were significantly higher than in males exposed to 6 mg/kg (*p* < 0.01) whereas no statistically significant differences were found in females.

##### Strain and sex interacting with dose × lag

The observed autocorrelations were higher and declined more gently in SHR/NCrl exposed to 1 mg/kg compared to unexposed SHR/NCrl and animals exposed to 3 mg/kg, whereas the steepest decline in autocorrelations was observed in SHR/NCrl exposed to 6 mg/kg. In WKY/NHsd, the observed autocorrelations decreased more gently across lag in animals exposed to 3 mg/kg compared the other exposure conditions. This strain × dose × lag interaction effect was statistically significant, F(4.79,295.58) = 7.26; p < 0.001. The analyses also showed a statistically significant sex × dose × lag interaction effect, F(4.79,295.58) = 2.81; p < 0.05. The autocorrelations declined more gently across lags in males exposed to 3 mg/kg compared to unexposed males or males exposed to 1 mg/kg, whereas the steepest decline was observed in males exposed to 6 mg/kg. In females, the autocorrelations across lags were similar in all exposure conditions except in the 1 mg/kg exposure condition where a more gentle decrease in autocorrelations across lags was observed.

## Discussion

The main aim of the study was to test effects of PCB 153 exposure on ADHD-like behaviors in the animal model of ADHD, the Spontaneously hypertensive rats (SHR/NCrl), and in Wistar Kyoto (WKY/NHsd) controls. Also, sex-specific effects and dose–response relations were explored. For this purpose, SHR/NCrl and WKY/NHsd rats, males and females, were exposed three times between PND 8 and 20 to PCB 153 at doses of 1, 3, or 6 mg/kg bodyweight or to corn oil only and then behaviorally tested from PND 37 to PND 64 for changes in stimulus control (sustained attention), number of lever-presses with IRTs > 0.67 s (activity), and number of lever-presses with IRTs < 0.67 s (impulsivity). Additionally, the animals were video-recorded during the operant task at PND 54 and 63, and amount of movement and the predictability of the movement pattern (autocorrelation of the animal’s position) in the operant chamber were analyzed.

The major short-term PCB 153 exposure effects include observations of: 1) No short-term effects of PCB exposure on stimulus control (attention) in SHR/NCrl. In WKY/NHsd, exposure to 1 mg/kg was associated with a short-term decrease in stimulus control that was more pronounced in males than in females. Still, a statistically significant difference was only found between the 1 mg/kg and 3 mg/kg WKY/NHsd exposure groups (Figure 1). 2) In SHR/NCrl, PCB exposure was associated with a short-term decrease in responses with long and short IRTs (activity and impulsivity, respectively) in animals exposed to 1 mg/kg. However, significant effects were only found for comparison of activity in the 1 mg/kg and 6 mg/kg SHR/NCrl exposure groups, and for comparisons of impulsivity in the 1 mg/kg with the 3 mg/kg or 6 mg/kg SHR/NCrl exposure groups. In contrast, activity was increased in WKY/NHsd exposed to 1 mg/kg and was significantly different from WKY/NHsd controls and animals exposed to 3 mg/kg, whereas no effects were found on impulsivity (Figures 2 and 3).

The major long-term PCB 153 exposure effects include observations of: 1) A long-term increase in stimulus control in male SHR/NCrl exposed to 1 mg/kg, whereas this effect was transient in SHR/NCrl females. Additionally, decreased stimulus control was observed in SHR/NCrl females exposed to 3 mg/kg and 6 mg/kg. However, significant differences were only found for comparisons of the 1 mg/kg SHR/NCrl exposure group with the 3 mg/kg or 6 mg/kg SHR/NCrl exposure groups. No long-term exposure effects on stimulus control were observed in WKY/NHsd (Figure 1). 2) A long-term decrease in activity in SHR/NCrl exposed to 1 mg/kg relative the other SHR/NCrl exposure conditions, and decreased impulsivity in SHR/NCrl exposed to 1 mg/kg compared to SHR/NCrl exposed to 3 mg/kg or 6 mg/kg. No long-term effects on these measures were observed in WKY/NHsd (Figures 2 and 3). 3) A long-term decrease in video-recorded movement in SHR/NCrl exposed to 1 mg/kg relative to SHR/NCrl exposed to 3 mg/kg or 6 mg/kg. However, exposure effects were mainly observed in SHR/NCrl females: Unexposed females moved significantly less than females exposed to 3 mg/kg, and females exposed to 1 mg/kg moved significantly less than females exposed to 3 mg/kg or 6 mg/kg. In contrast, no exposure effects on amount of movement were observed in WKY/NHs (Figure 4). 4) The autocorrelations of the position in the chamber tended to be higher in SHR/NCrl exposed to 1 mg/kg compared to unexposed controls. Also, the autocorrelations in SHR/NCrl exposed to 6 mg/kg and in SHR/NCrl females exposed to 3 mg/kg tended to be lower than in unexposed controls. The autocorrelations in unexposed WKY/NHsd males tended to be lower than in males exposed to 3 mg/kg and higher than in males exposed to 1 mg/kg, whereas no effects were observed in WKY/NHsd females. The statistical analyses showed significantly higher autocorrelations in SHR/NCrl exposed to 1 mg/kg compared to the 6 mg/kg exposure group. In contrast, the autocorrelations were significantly lower in WKY/NHsd exposed to 1 mg/kg compared to WKY/NHsd exposed to 3 mg/kg. Also, statistically significantly higher autocorrelations were found in males exposed to 3 mg/kg compared to males exposed to 6 mg/kg, whereas no significant differences were found in females (Figure 5).

The present findings further indicate that video-derived measures may supplement observations of lever pressing for the general purposes of testing behavior-altering effects of PCB 153 exposure (Figures 4 and 5). A previous study using an identical experimental procedure found a dissociation between video-recorded movement and rate of lever-pressing, suggesting that these measures tap into different behaviors [70]. A similar effect was found in the present study. PCB 153 exposure was associated with changes in autocorrelations of the animal’s position in the operant chamber although no changes were observed for lever-pressing, especially in the males. This dissociation can be seen by comparing Figures 1, 2, 3 (video-analyzed sessions are marked in red on the x-axes) with Figure 5 for doses 3 mg/kg and 6 mg/kg, and support the suggestion that two different aspects of behavior are measured. Generally, the predictability of the sequential movement pattern as analyzed by autocorrelations of the animal’s position in the test chamber shows that predictability is lower in SHR/NCrl, consistent with the dynamic developmental theory of ADHD and with findings in children with ADHD [30,45,46]. This finding is also consistent with studies of SHR/NCrl using other measures of reduced behavioral predictability (e.g. intra-individual variability, entropy) [71,72].

Taken together, our data show that exposure to PCB 153 produced a complex pattern of effects that depended on strain, sex, dose, time of testing, and the behavioral measure used. Generally, exposure produced long-lasting and larger behavioral changes in SHR/NCrl compared to WKY/NHsd controls, with mainly small and transient behavioral changes observed in the latter. Compared to unexposed controls and for many of the behavioral measures, the observed exposure effects of one dose were in the opposite direction to that observed following a higher dose, especially for SHR/NCrl. For several of the post hoc analyses, statistically significant differences were found only between two or more doses, whereas few significant effects were found between one individual dose and the unexposed control condition. This lack of a statistically significant difference between the exposed and unexposed groups complicates the interpretation of the results. Still, the data show a consistent pattern of effects across all behavioral measures that seems an unlikely chance occurrence. Overall, the data indicate a non-linear dose–response relationship with opposite effects of the doses tested, and where the effects reached the level of significance only when these opposite effects were compared.

The diversity of experimental procedures used in a number of published PCB studies precludes a direct comparison with our findings. Possibly, most relevant to the present results due to similarities in experimental procedure are the studies by Holene et al. which observed a long-lasting decrease in stimulus control and an increase in activity and responses with short IRTs in male, but not female, offspring of DA/OLA/HSD females mated with Lewis rats and gavage-fed 5 mg/kg PCB 153 every second day from PND 3 to 13 [73,74]. Comparable effects were found by Berger et al. in adult male Sprague–Dawley rats exposed to low doses of the PCB mixture Aroclor 1248 (30 g daily portions of rodent diet supplemented with 1 ml corn oil containing 0.5 μg/g Aroclor 1248) in the diet during puberty [75]. In the present study, similar behavioral patterns following PCB exposure were observed in female SHR/NCrl exposed to 3 and 6 mg/kg, and partly in male WKY/NHsd following exposure to 1 mg/kg (Figures 1, 2, 3). The combined result patterns indicate that a number of variables interact with PCB exposure, and that the neurotoxic effects on the neural system may differ across strain, sex and dosing regimen.

### Distinct durations of PCB 153 exposure effects in SHR/NCrl and WKY/NHsd controls

Exposure to PCB 153 produced behavioral changes in SHR/NCrl lasting six week following the final PCB exposure as opposed to the mainly short-lasting behavioral changes observed in the WKY/NHsd controls. Exposure to 1 mg/kg was associated with increased stimulus control and decreased lever pressing in SHR/NCrl, while the same dosage tended to produce temporary behavioral changes in the opposite direction in WKY/NHsd (Figures 1, 2, 3). The pattern of behavioral changes observed in SHR/NCrl following exposure to 1 mg/kg was similar to the pattern of behavioral changes observed in a previous study of outbred male WKY/NTac exposed to 10 mg/kg using an identical experimental procedure [61]. Combined, the findings suggest an increased behavioral sensitivity to PCB 153 exposure in the SHR/NCrl as compared to WKY/NHsd controls.

The mechanisms involved in the PCB 153-induced effects remain unclear in both rat strains. PCB 153 exposure may produce more behavioral changes in SHR/NCrl because the toxic substance acts synergistically with dysfunctioning transmitter systems in this rat strain. This is consistent with the known effects of PCB exposure on transmitter systems like dopamine and noradrenalin and findings suggesting that the behavioral alterations in SHR/NCrl are driven by an imbalance in these transmitter systems [48,52-56]. Thus, the observed effects in SHR/NCrl on reinforcer-controlled lever-pressing may be produced by PCB exposure effects on the dopamine system which is central to the reinforcement process [42,43]. The video-derived measures partly reflect reinforcer-controlled behaviors, but also other behaviors occurring during testing like locomotion, exploration, and grooming. Studies have linked exploratory activity to the mesolimbic and mesocortical dopamine systems and to increased glutamatergic signal transmission, which are transmitter systems affected by PCB exposure [16,76,77].

The involvement of dopamine and other catecholamines in the behavioral changes observed in SHR following PCB exposure is supported by a study of similarities in RNA gene-expression levels between unexposed SHR/NCrl and Sprague–Dawley rats (NTac:SD) exposed to a 1:1 mixture of Aroclor 1254/1260 from gestational day 5 to 19 at a dose of 4.0 μg/g body weight [14]. The study found that while many gene expression levels were different in the unexposed SHR/NCrl and the PCB-exposed Sprague–Dawley rats, similarities were found in genes related to dopamine transmission in the striatum and to the COMT gene (catechol-O-methyltransferase) coding for methylation of catecholamine neurotransmitters, including dopamine [14]. However, the authors concluded that these similarities could be short-term or long-term as well as compensatory [14]. This complexity of relating PCB exposure to gene expression changes is further illustrated by a second study which found dysregulated expression of many genes in Sprague–Dawley rats following lactational, but not following gestational, exposure to the PCB mixture Aroclor 1254, and that gene expression levels also depended on brain region as well as time of observation [78]. An important implication of the present findings is that when studying animals exposed to PCBs as an environmental model of ADHD, behavioral data should be included to ensure that the animals tested display the ADHD-like behavioral phenotype. Behavior is the most important validation criterion for animal models of ADHD [47-49,51]. Indeed, the present data suggest that PCB exposure in some cases may *reduce* ADHD-like behavior, as observed in SHR/NCrl females and in WKY/NHsd controls following exposure to 1 mg/kg. Hence, behavioral data is imperative to establish a relation between changes in gene expression and ADHD-related behavior.

Furthermore, it is possible that PCB exposure interacts with a neurodevelopmental time-course that is different in SHR/NCrl and WKY/NHsd controls. PCB 153 exposure effects appear to be age-dependent, with exposure effects on motor coordination and activity level in Wistar rats following different time-courses in males and females, thereby accentuating the relation between neurodevelopment, time of exposure and behavioral exposure effects [79,80]. Further, studies show that maturation is delayed in SHR relative to WKY and other strains [81,82]. The increased sensitivity to PCB 153 exposure in SHR/NCrl may be caused by exposure effects interacting with the neurodevelopmental time-course through specific effects on cellular mechanisms underlying learning and memory. The N-methyl-D-aspartate receptor (NMDAR) is important for long-term potentiation (LTP) which is proposed to be a cellular mechanism for learning and memory [83,84]. PCB exposure impairs NMDA receptor-mediated signaling, and has been reported to reduce LTP in the CA1 region of the hippocampus in rats [85-90]. The NMDARs are composed of NR1A and NR2B subunits that determine the receptor’s functional properties [91-93]. During neurodevelopment, there is a shift in the relative number of NR1 to NR2 subunits, where a predominance of NR2B subunits is characteristic of the early developmental stages of these synapses. Lehohla et al. (2004) found impaired NMDA receptor function in prefrontal cortical slices in SHR/NCrl [94]. Jensen et al. showed that NMDAR-dependent LTP in hippocampal CA3-to-CA1 synapses was significantly reduced in SHR/NCrl by the NR2B-specific blocker CP-101,606, suggesting a delay or disturbance in neurodevelopmental maturation in SHR/NCrl relative to WKY/NHsd controls [95]. Thus, slowed or hampered neurodevelopmental maturation in SHR/NCrl may interact with (time of) PCB exposure and produce behavioral effects that are different or more severe in SHR/NCrl than in WKY/NHsd controls.

### Sex-specific effects

In general, PCB 153 exposure of SHR/NCrl was associated with more behavioral changes in females than males although not all analyses reached the conventional level of significance. In contrast, there was a tendency for opposite effects in WKY/NHsd controls. The analyses showed statistically significantly sex × dose interaction effects for measures of impulsivity, amount of video-recorded movement and autocorrelations of position during testing (Figures 3, 4, 5). For lever pressing, a serpentine-shaped dose–response relationship was observed in the females, whereas this relationship was L-shaped in the males. Additionally, a significant difference between males and females in amount of movement was found following exposure to 3 mg/kg as well as specific effects of doses in SHR/NCrl females but not in SHR/NCrl males. Thus, the overall findings indicate that PCB 153 exposure has sex-specific effects that additionally may depend on strain.

PCB exposure has been shown to disrupt endocrine functions and sex hormone levels which play an important role in the development of the nervous system [16,17,96]. The present data suggest that PCB exposure interacts differently with these systems in SHR/NCrl and WKY/NHsd, but this needs further investigation. However, the present findings are in agreement with other studies showing sex-specific effects of PCB 153 or Aroclor 1254 exposure in rats, e.g. [73,74,80,97-101]. This is also consistent with findings in humans indicating that PCB exposure has gender-specific effects and is more detrimental in women [102].

### The dose–response relation

The present data frequently suggest a non-linear dose–response relation where small doses of PCB can have the opposite behavioral effects compared to high doses. This is most apparent for impulsivity and the video-derived measures (Figures 3, 4, 5), and was most prominently observed in female SHR/NCrl but is also to some extent observed in SHR/NCrl and WKY/NHsd males. In a previous study using an identical behavioral procedure, WKY/NTac males exposed to 10 mg/kg PCB 153 showed a pattern of behavioral changes opposite to the behavioral changes observed in WKY/NHsd males exposed to 1 mg/kg in the present study [61]. Studying Aroclor 1254 exposure effects in rats, Nishida et al. and Kodavanti et al. observed dose-dependent reductions in activity following PCB exposure whereas increased activity was found by Berger et al. [75,103,104]. Taken together, these results indicate a non-linear relationship between dose and behavioral changes following PCB exposure, and possibly also that the dose–response relationships may be different across brain regions and measures [105-108]. A simple explanation of a non-linear behavioral dose–response relation is that low-dose exposure produces specific and limited effects in the nervous system while more widespread, systemic effects are produced by high doses. Additionally, compensatory mechanisms initiated by the nervous system following neurotoxic assault may depend on dose and, hence, produce non-linear behavioral effects. However, PCB exposure may also produce non-linear behavioral dose–response effects through e.g. changes in the dopamine system, as illustrated by the Heinzel and Dresler et al. [109] study finding that depending on dopamine D4 receptor genotype, performance on a Go-NoGo task followed an inverted U-shape with increasing dopamine levels [109].

### PCB exposure as an environmental risk factor in ADHD

PCB exposure has been proposed as an environmental risk factor that either alone or in interaction with a genetic vulnerability can produce ADHD [10-15]. This association is supported by findings linking ADHD to dopamine and noradrenalin dysfunction, both of which are transmitter systems affected by PCB exposure [3,30,41]. The present data indicate that the degree and quality of the behavioral changes following PCB 153 exposure depend on several factors including strain, sex and dose. SHR/NCrl exposed to 1 mg/kg showed *improved* stimulus control and *reduced* lever-directed activity, while doses of 3 and 6 mg/kg tended to exacerbate the ADHD-like behaviors characteristic of SHR/NCrl. Also, the present findings indicate that PCB 153 exposure effects are more pronounced in SHR/NCrl females than in SHR/NCrl males. For the WKY/NHsd controls, PCB 153 exposure did not unequivocally produce the full pattern of behaviors observed in SHR/NCrl. Different results may be obtained with higher doses, and should be tested. However, a previous study of Wistar Kyoto (WKY/NTac) exposed to 10 mg/kg at PND 8, 14, and 20 using an identical behavioral procedure observed *reduced* activity levels following exposure, suggesting that high-dose exposure to PCB 153 does not produce the full SHR/NCrl behavioral phenotype in control animals in the presently used experimental procedure [61]. To the extent the present findings can be generalized to ADHD, it seems that PCB 153 exposure can moderate or exacerbate ADHD behaviors in genetically vulnerable individuals depending on amount of exposure, and possibly particularly in females, while having only small and mainly transient effects in typically developing children.

## Conclusion

PCB 153 exposure was associated with pronounced and long-lasting behavioral changes in SHR/NCrl, suggesting greater behavioral sensitivity to PCB exposure in these animals as compared to WKY/NHsd controls. PCB 153 exposure did not unequivocally aggravate ADHD-like behaviors in SHR/NCrl but depended on dose where 1 mg/kg tended to reduce ADHD-like behaviors and produce opposite behavioral effects compared to 3 mg/kg and 6 mg/kg, especially in the SHR/NCrl females. For the WKY/NHsd controls and for the three doses tested, PCB 153 exposure did not produce the full range of ADHD-like behaviors which were observed in unexposed SHR/NCrl, but produced a few specific behavioral changes in males only.

To the extent the present findings can be generalized to human ADHD, exposure effects of PCB 153 on ADHD behavior depend on amount of exposure: Low doses moderate and high doses aggravate ADHD symptoms in genetically vulnerable individuals. In normal controls, exposure does not seem to constitute an environmental risk factor for developing ADHD, but can produce specific behavioral changes.

At a general level and consistent with previous findings, the present findings suggest that behavioral changes can be related to dose in an non-linear fashion, and that exposure to PCB 153 interacts with several variables, including strain, sex, dose, and time of testing.

## Competing interests

The authors declare that they have no competing interests.

## Authors’ contributions

EBJ participated in designing the study, and had the main responsibility for analyzing the data and drafting the manuscript. FF participated in designing the study, made the PCB-solutions used in the study, and helped drafting the manuscript. PLL participated in designing the study, had the main responsibility for animal breeding and welfare, administered the PCBs, and helped drafting the manuscript. SIV participated in designing the study and helped writing the manuscript. NEB had with EBJ the main responsibility for the data analyses and helped drafting the manuscript. GW was the main organizer of animal testing and data collection and performed preliminary data analyses. TS was the study coordinator and participated in the design of the study, but passed away before the finishing of the manuscript. All authors except TS read and approved the final manuscript.
